# Factors contributing to resilience amongst adolescent girls in artisanal and small-scale mining communities in Ghana and Uganda

**DOI:** 10.1080/21642850.2026.2668157

**Published:** 2026-05-06

**Authors:** Hanna Chidwick, Betty Kwagala, Asiimwe John Bosco, Nowshin Nahela, Lydia Kapiriri

**Affiliations:** a Department of Health, Aging & Society, McMaster University, Hamilton, Canada; b Department of Population Studies, Makerere University, Kampala, Uganda

**Keywords:** Artisanal and small-scale mining, adolescent, resilience, health, economic

## Abstract

**Background:**

Resilience is critical to the health and well-being of last-mile adolescent girls and young women (AGYW) who work in artisanal and small-scale mining (ASM) communities. The COVID-19 pandemic exacerbated the health and economic challenges that AGYW in ASM communities experience. Despite a growing focus and discussion of adolescent resilience in the literature, there is a continued gap in information on the experience of last-mile or hard-to-reach adolescents, such as those in ASM communities, and their resilience. This paper aims to understand adolescent resilience in ASM communities in Uganda and Ghana and identify contributing factors.

**Methods:**

This study employed quantitative analysis of survey responses from 1618 AGYW in Ugandan and Ghanaian ASM communities. Survey questions focused on resilience and were adapted from the Brief Resilience Scale (BRS). Responses were analyzed (descriptive, bivariate, logistics regression) using STATA and BRS scoring to indicate low and normal/high resilience.

**Results:**

Across AGYW in both Ghana and Uganda, an average of 57% had a normal/high resilience score and 43% had a low resilience score. Variables that were significantly related to normal/high resilience (p value equal to or less than 0.05) included residence in Ghana, AGYW 18 years or above, completed secondary education, were migrants, were employed by their parents or other relatives, received fair payments, had savings, drank alcohol, did not experience physical violence or a combination of physical and sexual violence, and had monthly incomes higher than $70.

**Conclusions:**

Findings correlate with existing literature on resilience amongst adolescents. Targeted resilience interventions with the AGYW in ASM have the potential to break cycles of vulnerability and should focus on savings and financial literacy, along with advocacy for mitigating sexual and gender-based violence and enhancing girls' rights to continue to build resilience. Long-term multi-stakeholder collaborations are key to building resilience within ASM communities.

## Introduction

Resilience is critical to the health and well-being of last-mile adolescent girls and young women (AGYW) who work in artisanal and small-scale mining (ASM) communities. AGYW working in ASM experience various health and economic challenges with limited access to external support, thus requiring self-reliance and resilience (Davison and Bartels, 2021; Nowshin and Kapiriri, 2022; Pedrajas & Choritz, 2016). Ghana and Uganda are home to last-mile ASM communities that experience these exacerbated challenges (Kwagala et al., 2025; Stemn et al., 2021). ASM represents a significant source of livelihood for millions of people in resource-rich developing countries, including Ghana and Uganda, and remains an important contributor to employment and poverty alleviation (Amoako et al., 2023; Hilson, 2016; Jackline, 2022; Landrigan et al., 2022). In addition to direct employment, the sector provides indirect employment to millions in sub-Saharan Africa (Amoako et al., 2023). Hilson (2016) points out that ASM in sub-Saharan African countries can be traced back to the ‘region's deeply rooted rural household diversification patterns’ (p.10). Not only is ASM a way to reduce risk by branching out to the non-farm economy, but the links between agriculture and ASM also provide a means for greater wealth creation for households (Hilson, 2016). Ghana and Uganda are among mineral-rich countries where ASM is prevalent, and the combined total of workers directly involved in ASM in these two countries exceeds 1.2 million (Hilson, 2016; Jackline, 2022). Despite efforts towards formalisation, much of the ASM sector remains unregulated in Ghana and Uganda due to a combination of complex factors (Jackline, 2022; Kumah, 2022). Licensing complexities and costs have been cited as a key concern in the case of both countries (GEF & UNEP, 2025; Hilson & Maconachie, 2020a). A bias toward large-scale mining companies, lack of political interest to develop an appropriate legal framework, and rent-seeking elements have been identified as barriers to formalisation in Ghana (Hilson & Maconachie, 2020a). While these factors are also relevant to Uganda, other challenges of formalisation in Uganda include miners' lack of access to capital required to invest in sustainable mining engineering, their distrust towards the government, and difficulties in monitoring small-scale mining (compared to large-scale companies) (GEF & UNEP, 2025; Siegel & Veiga, 2009).

The ASM sector in Uganda provides work for 90,000 women (Mpagi et al., 2017) and for over 500,000 women in Ghana, representing 50% of the workforce (McQuilken & Hilson, 2016; Osei et al., 2021). A significant portion of these women are between the ages of 10–24 and experience multiple gendered, socio-economic, and health vulnerabilities that affect their well-being and resilience during crises (Buss et al., 2017; Danielsen & Hinton, 2020; Jenkins, 2014). Despite the diverse activities women undertake, there exists limited information on women's involvement in the ASM sector, which represents a significant knowledge gap (Jackline, 2022). Reliable information is needed in this regard to enact appropriate policies, implement interventions, and address the needs of female miners (Jackline, 2022; Ofosu et al., 2024). Moreover, research is scarce on the resilience of AGYW in ASM communities which is crucial for understanding the well-being of this last-mile population who are at risk of experiencing multiple intersecting vulnerabilities (Kwagala et al., 2025).

### ASM in the context of the SDGs

The sustainable development goals (SDGs) were established to provide a framework for monitoring global initiatives aimed at sustainable development in social, health, economic, and ecological sectors (United Nations, 2020). Central to this framework is the principle of ‘leaving no one behind’ (Hilson & Maconachie, 2020b). Nonetheless, the original design of the SDGs overlooked ASM (Hilson & Maconachie, 2020b). This represents a significant omission considering the sector's contribution to poverty alleviation (SDG 1) and economic growth (SDG 8) by providing employment and income to millions (Laing & Moonsammy, 2021). Notwithstanding these benefits, it often operates informally, and the utilisation of toxic substances, including mercury and cyanide, poses substantial health and environmental challenges, associated with SDGs 3, 13, 14, and 15 (Cheng et al., 2023; Clifford, 2022). The absence of formal governance structures also exacerbates social problems, such as child labour and gender inequality, where women face restricted access to economic and social opportunities (Tekinbas, 2022; Yakovleva et al., 2022). This undermines targets of gender equality (SDG 5), decent work (SDG 8), and quality education (SDG 4). Although the economic importance of ASM has been recognised in sub-Saharan African countries, a lack of understanding of its complexities has impeded effective policy integration (Hilson & Maconachie, 2020b). Top-down policymaking approaches further hinder collaborative governance needed for achieving the SDGs (Hilson, 2025). Mapping ASM's impacts to the SDGs, utilising country-specific case studies, can help in formulating effective, contextually relevant policies in sub-Saharan African countries (Laing & Moonsammy, 2021).

### The gendered dimensions of ASM

Discussions on women's involvement in ASM began in the 1990s and 2000s (Hinton et al., 2003; Labonne, 1996; Nandi & Aich, 1996; Yakovleva, 2007), with recent literature illuminating the catalysts of women's engagement, its significance for economic empowerment, and the need for the sector's formalisation (Arthur-Holmes & Abrefa Busia, 2022a; Buss et al., 2022; Hilson et al., 2018). In sub-Saharan African countries, approximately 40–50% of ASM's workforce constitute women (Hinton et al., 2003). For many women, ASM provides an essential source of income amid poverty, restricted formal employment, and declining agricultural yields (McQuilken & Hilson, 2016; Tekinbas, 2022; Yakovleva, 2007). The gendered dimensions of ASM are evident in the differences in the tasks that men and women carry out. The lucrative tasks deemed ‘masculine’ are taken up by men, while the work typically undertaken by women (like cleaning, transporting, and cooking) is considered comparatively less valuable which contributes to the power and dominance men enjoy in the sector (Byemba, 2020; Danielsen & Hinton, 2020). An example of a lucrative opportunity is digging hard-rock deposits which requires teams comprised of multiple workers and approvals from the concerned authority (Danielsen & Hinton, 2020). Participation in such teams requires networking and consistent attendance, and women's irregular availability due to the pressures of other responsibilities may make them undesirable for tasks that require teamwork (Danielsen & Hinton, 2020). In other cases, the costs of joining (such as cattle or money) can act as deterrents for women (Perks et al., 2015). Nonetheless, women's involvement is also affected by caregiving and reproductive responsibilities (Buss et al., 2017). Gender norms continue to impede women's access to lucrative positions within the sector. For example, in Ghana, illiteracy and technical skills deficiency, along with household responsibilities, limit their opportunities for higher-paying roles (Yakovleva, 2007). The absence of legal and economic rights often results in women's labour being devalued or sometimes unpaid, especially in roles considered menial eg, sand transportation (Arthur-Holmes & Abrefa Busia, 2021). Consequently, this vulnerable group has to manoeuvre through intricate social hierarchies and gendered power dynamics to advocate for fair remuneration (Arthur-Holmes et al., 2023a). The limited support on mining sites, e.g. the lack of childcare facilities for children who accompany their mothers to work, exposes both parties to serious health risks and physical hazards (Arthur-Holmes & Abrefa Busia, 2021). These occupational health risks encountered by women are compounded by inadequate safety protocols and protective apparel (Arthur-Holmes & Abrefa Busia, 2022b; Stemn et al., 2021). Furthermore, the scarcity of economic opportunities in rural communities, coupled with financial hardships and the absence of gender-sensitive protections in ASM, also compels young and migrant women to engage in transactional sex as a means of livelihood (Arthur-Holmes et al., 2025) which increases the risk of poor sexual health outcomes. The results of a cross-sectional survey conducted in ASM communities in Uganda showed that approximately half of AGYW had a self-reported STI or symptoms of STIs which was much higher than the national prevalence (Kwagala et al., 2025). Notwithstanding these complex challenges, numerous women persist in engaging in ASM due to its comparatively stable income relative to other options (Hilson et al., 2018). This paradox necessitates urgent interventions that not only alleviate occupational health risks and tackle gender disparities but also address the intricate socioeconomic factors influencing women's involvement in ASM. AGYW represent a last-mile population who experience intersecting vulnerabilities associated with age, gender, and socioeconomic status (Chidwick et al., 2023; Kwagala et al., 2025). For example, some pregnant AGYW, due to the lack of support from their family or partner, get involved in ASM to be able to afford basic necessities (Arthur-Holmes et al., 2023b). The strenuous work pregnant AGYW carry out reflects the intersectional nature of health risks associated with ASM which is already characterised as high risk.

### The impact of COVID-19 on ASM communities

The COVID-19 pandemic exposed and intensified pre-existing structural vulnerabilities and inequalities in ASM communities in sub-Saharan African countries (Calvimontes et al., 2020; Hilson et al., 2021; Thierens & Mawala, 2020). Mobility restrictions, supply chain interruptions, and falling mineral prices significantly affected livelihoods (Laing, 2020). Lockdowns resulted in substantial income loss for ASM workers, impacting their capacity to meet their basic needs. Rising production costs, restricted financial access, and poor working conditions further aggravated these economic challenges (Muthuri et al., 2021). Food insecurity in mining communities also worsened due to escalating staple food prices and declining incomes (Perks & Schneck, 2021). Additionally, the pandemic revealed gendered impacts, where women disproportionately bore childcare and homeschooling responsibilities amid school closures. In Ghana, the suspension of school feeding programmes shifted food responsibilities to mothers, amplifying financial challenges and the risk of child hunger in already vulnerable households (Muthuri et al., 2021). Research conducted in Zimbabwe, Uganda, and Kenya indicated that women experienced elevated rates of unemployment during the pandemic (Lyster & Singo, 2020). In Kenya, restrictions on mining activities due to lockdowns compelled numerous women to engage in nocturnal mining, heightening their susceptibility to gender-based violence (Kenya Land Alliance, 2021). Poor working conditions and the absence of social protections exacerbated safety and health risks, especially for women (Muthuri et al., 2021). Adolescent girls, who are ‘forgotten’ in times of crises, were faced with the increased risk of sexual exploitation and sexual and gender-based violence in the COVID-19 pandemic, especially those in resource-limited settings (Murewanhema, 2020). Despite these challenges, some communities demonstrated resilience through self-organised responses to the pandemic's crises (Calvimontes et al., 2020). These dynamics highlight the need for equitable, gender-sensitive reforms and inclusive ASM policies to enhance social protection and incorporate the sector into comprehensive development strategies (Hilson et al., 2021).

### Understanding individual resilience

To address increasing crises related to health, climate, and economy, individual and community resilience has become an increasing focus of research and projects including the SDGs (Bahadur and Lovell, 2015; Chen et al., 2022; Laurenzi et al., 2021; Rother et al., 2020; UN Sustainable Development Group, 2022; United Nations General Assembly, 2015). Resilience has been defined as the ability to bounce back from challenges (Levine, 2003; Namy et al., 2017; Smith et al., 2008), and in this study, is considered in terms of flexibility, inner-strengths, capacity (including organisational resources), minimising casualties, adapting, growing, and flourishing (Chidwick et al., 2023). For individuals, resilience has been discussed as an important aspect of adolescence especially—a time of growth and development (Namy et al., 2017). There are many factors that contribute to resilience including personal characteristics such as attachment, health, productivity, and optimism, along with external factors such as environment, engagement with nurturing adults, and schooling (Bandeira and Graham, 2023; Levine, 2003). From a life course perspective, adolescence and childhood are critical periods for building resilience through fostering personal characteristics and positive environments. Adverse experiences in childhood and adolescence, such as violence, if not addressed, often result in mental and physical health challenges, with subsequent negative impact on their education. It also increases the likelihood of their perpetrating violence in adulthood (Namy et al., 2017). Despite these challenges, there are limited programmes and services to support adolescents' resilience and mental health support in Ghana and Uganda.

Literature shows that to enhance the resilience of adolescents in sub-Saharan African countries, including Ghana and Uganda, there is a need for context-specific, multi-level, and community-involved interventions through fostering both individual and environmental resources (Bandeira and Graham, 2023; Groves et al., 2022; Kabiru et al., 2012; Khanlou & Wray, 2014). Most of the existing literature on adolescent resilience has focused on child and adolescent health in school settings/school-based programmes (Dray et al., 2015; Theron, 2020; Twum-Antwi et al., 2020). Recent scholarship has discussed adolescent resilience in terms of the COVID-19 pandemic, given the significant impact it has had on adolescent mental and physical health, along with their relationships (Bischops et al., 2022; Branje & Morris, 2021; Garagiola et al., 2022; O’Brien et al., 2021; Shi and Zhao, 2022; Yusuf et al., 2022). Mhaka-Mutepfa et al. (2019) discuss the importance of collaboration between community leaders including religious and non-religious leaders, to foster adolescent resilience, especially with the most vulnerable adolescents. Levine (2003) notes that investing in programmes, such as family-centred, school-centred, and neighbourhood-centred programmes and therapy, that support resilience is key to enhancing early childhood and adolescent resilience. The Uganda and Ghana literature on adolescent resilience has explored factors that contribute to resilience, including sports-based programmes (Abonga & Brown, 2022); social relationships, friendships and access to interpersonal resources (Abonga & Brown, 2022; Asante, 2019; Eggum et al., 2011; Gyan et al., 2017; Tutu, 2013); spirituality and gaining hope through belief in God (Asante, 2019; Eggum et al., 2011; Mhaka-Mutepfa & Maundeni, 2019); and, community-based programming from organisations and governments to build skills (Asante, 2019).

Despite a growing focus and discussion of adolescent resilience in the literature, there is a continued gap in information on the experience of last-mile or hard-to-reach adolescents such as those in ASM communities, and their resilience (Chidwick et al., 2023). Existing literature about ASM has focused on the intersections of economic sustainability and health (Buss & Rutherford, 2020; Buss et al., 2017; Hilson & Osei, 2014; Hilson et al., 2021; Hilson, 2009; Jenkins, 2014) and the mental health of mine workers (Asare-Doku et al., 2022; Cambaco and Cossa, 2024; Matamala Pizarro & Aguayo Fuenzalida, 2021). This paper fills this gap in the literature by providing insights into the factors that contribute to the resilience of last-mile AGYW living in ASM communities.

This paper aims to assess the resilience of AGYW living in ASM communities in Uganda and Ghana and to identify the factors that contribute to their resilience. The findings can inform the development of interventions and programmes for strengthening ASM- AGYW's resilience.

## Materials and methods

### Study setting

This study was conducted in Uganda (Busia, Namayingo, Kassanda, Mubende) and Ghana (Banda Nkwanta, Bamboi, and Chingakrom). While the ASM sector in Ghana is more established compared to the ASM sector in Uganda. Hence, the two countries presented an opportunity for South-to-South learning. ASM communities tend to be remote, hard to reach, and lack infrastructure. The project's goal was to shed light on a last-mile population, namely the adolescent girls and young women (AGYW) in ASM communities. AGYW in these communities are marginalised, lacking access to social, economic, and health resources. They also tend to be excluded from the national statistics and research.

### Data collection

As part of a larger mixed-methods study on adolescent resilience in ASM communities (Author, n.d.) this paper used quantitative analysis of survey responses from adolescent girls and young women. The study was approved by the research ethics committees in three countries—Uganda, Ghana, and Canada. All participation was voluntary, and all participants provided informed consent.

We surveyed 1618 adolescent girls and young women aged 20–24 years (809 per country), who experience exacerbated vulnerability and challenges in ASM communities. We used Yamane's (Yamane, 1973) formula to determine the sample size, taking into consideration the design effect of 2 and the anticipated response rate of 98%. We included young women aged 20–24 because most adolescent young women involved in ASM communities were within that range and had started work as adolescents. Adolescent girls were eligible to participate if they either directly worked in the mines or provided services to ASM workers (i.e. food provision, etc.). Data was collected through the Kobo Collect tool using a pre-tested survey questionnaire, which was translated into the relevant local languages. The questions, among others, focused on resilience which were adapted from the Brief Resilience Scale (BRS) (Smith et al., 2008) (see Table 1).

**Table 1.** Adapted brief resilience scale (Smith et al., 2008).

	Brief resilience scale (BRS)	Strongly agree	Agree	Neutral	Disagree	Strongly disagree
1.	I usually bounce back/get back to normal quickly after hard economic times	5	4	3	2	1
2.	If I don't earn mining-related work, I have other means of sustaining myself and family	5	4	3	2	1
3.	I have a hard time making it through stressful events	1	2	3	4	5
4.	It does not take me long to recover from a stressful event	5	4	3	2	1
5.	It is hard for me to bounce back when something bad happens	1	2	3	4	5
6.	I usually come through difficult times with little trouble	5	4	3	2	1
7.	I tend to take a long time to get over setbacks in my life.	1	2	3	4	5
8.	I tend to bounce back quickly, learn from the situation, and transform after hard times	5	4	3	2	1

Survey responses were synthesised and analysed, using STATA, based on Table 1. First, we generated the respective frequencies for each item in the scale. Next, we calculated each participant's BRS score following the adapted BRS scoring chart in Table 2. Scores were calculated by adding the value of the responses for each participant (1–5) for all 8 items and subsequently dividing the sum by the total number of questions (8) for the final score for each participant. We then used these composite scores to complete univariate, bivariate, and the logistic regression analysis. Independent variables included age, region, country, marital status, employment, income, etc.

**Table 2.** BRS scoring.

BRS score	Interpretation
1.00–2.99	Low resilience
3.00–5.00	Normal to high resilience

## Results

### Demographic information

Results show that on average, 70% of participants in Uganda and Ghana were above the age of 18 years and 63% were single and had never been married. In terms of education, an average of 36% had completed secondary education across the two countries. The majority of participants (64%) had migrated to work in the ASM communities. While almost all (97%) AGYW engaged in mine-related work from time to time, 36% of adolescents worked directly in the mines most of the time, and the rest 64% predominantly worked in the service sector (see Table 3). The majority of AGYW (61%) indicated they were employed by another individual and reported that they were not paid fairly at work (56%). About 44% of adolescents within the ASM communities indicated that they earn less than $70 USD per month. Despite low earnings, 56% of AGYW indicated that they had savings, but the majority (82%) did not belong to any kind of savings group.

**Table 3.** Overview of employment, payment and savings.

Survey variable	% in Uganda	% in Ghana	Average % across Ghana and Uganda
**Type of work**			
1. Directly works in	27.5	44.3	35.9
2. Services & others	72.5	55.7	64.1
**Type of employer**			
1. Self-employed	42.7	18.4	30.6
2. Employed by a non-related individual	50.4	72.4	61.4
3. Employed by parents, relatives	6.9	9.2	8.0
**Perceived fairness of payment**			
1. Yes	32.1	56.3	44.2
2. No	67.9	43.7	55.8
**Has savings**			
0. No	40.9	46.7	43.8
1. Yes	59.1	53.3	56.2
**Belongs to a savings group**			
0. No	73.6	89.7	81.6
1. Yes	26.4	10.3	18.4
**Monthly earnings**			
0. Less than $70 USD	46.5	42.7	44.6
1. Greater than or equal to $70 USD	37.9	52	44.9
No earning	15.6	5.3	10.4

In addition to work-related demographics, average results from both countries show that 13% of participants had experienced physical violence (22% in Uganda and 4% in Ghana) and 8% had experienced sexual violence (12% in Uganda and 5% in Ghana) during the COVID-19 pandemic. In both countries, an average of 15% (19% in Uganda and 12% in Ghana) indicated that they drink alcohol.

### Resilience analysis

Table 4 shows the resilience status of participants, where an average of 57% of adolescents had a normal to high resilience score and 43% of adolescents had a low resilience score across Ghana and Uganda.

**Table 4.** Brief resilience scale—overall results.

Resilience score	% in Uganda	% in Ghana	Average % across Ghana and Uganda
1.00–2.99, low resilience	50.5	35.2	42.8
3.00–5.00, normal to high resilience	49.5	64.8	57.2

Table 5 indicates that resilience varied significantly with all independent factors with the exception of type of work, whether AGYW belonged to a savings group, whether AGYW worked during the lockdown, and sexual violence. Variables that were significantly related to normal/high resilience (*p* value equal to or less than 0.05) included adolescent girls in Ghana, those who were 18 years or above, had a secondary education, were migrants, were employed by their parents or other relatives, received fair payments, had savings, drank alcohol, did not experience physical violence or a combination of physical and sexual violence, and had monthly incomes higher than $70.

**Table 5.** BRS bivariate analysis.

Variable	Low resilience	Normal to high resilience	Row totals	*P* value
Country of residence				**0.000**
1. Uganda	50.5	49.5	810	
2. Ghana	35.2	64.9	808	
Age				**0.002**
1. <18 years	49.2	50.8	441	
2. 18+ years	40.4	59.6	1177	
Marital status				0.728
1. Single never married	43.6	56.4	1026	
2. Married	41.7	58.3	480	
3. Previously married	41.7	58.3	112	
Religion				**0.000**
1. Christians	45.9	54.1	1,219	
2. Muslims	33.4	66.6	341	
Others	32.8	67.2	58	
Migration status				**0.001**
0. Migrated	42.8	57.2	1030	
1. Not migrated	48.3	51.7	588	
Education level				**0.000**
1. None	40.1	59.9	304	
2. Primary	50.1	49.9	731	
3. Secondary	35.2	64.8	583	
Type of work				0.256
1. Directly works in	41.0	59.0	581	
2. Services & others	43.9	56.1	1037	
Type of employer				**0.001**
1. Self-employed	49.5	50.5	495	
2. Employed	40.2	59.8	993	
3. Parents and other relatives	37.7	62.3	130	
Perceived fairness of payment				**0.000**
1. Yes	33.6	66.4	715	
2. No	50.2	49.8	903	
Has savings				**0.000**
0. No	49.0	51.0	708	
1. Yes	38.0	62.0	910	
Belongs to a savings group				0.350
0. No	43.4	56.6	1321	
1. Yes	40.4	59.6	297	
Worked during the lockdown				0.581
0. No	43.5	56.5	795	
1. Yes	42.2	57.8	823	
Uses alcohol and substances				**0.000**
0. No	45.0	55.0	1369	
1. Yes	30.9	69.1	249	
Physical violence				**0.000**
0. Not experienced	41.0	58.9	1404	
1. Experienced violence	54.2	45.8	214	
Sexual violence				0.053
0. Not experienced	42.1	57.9	1484	
1. Experienced violence	50.8	49.3	134	
Physical and/or sexual violence				**0.000**
0. Not experienced	40.9	59.1	1356	
1. Experienced violence	53.1	47.0	262	
Monthly earnings				**0.000**
0. Less than 70$	47.8	52.2	722	
1. > = 70$	37.4	62.6	727	
No earning	45.0	55.0	169	

Logistic regression analysis, outlined in Table 6, further shows the key variables that correlated with normal to high resilience.

**Table 6.** Logistic regression analysis of the determinants of resilience.

Variable	Odds Ratio	*P* > z	95% Conf.	95% Conf.
**Country**				
1. Uganda (RC)				
2. Ghana	**1.56**	0.001*	1.213147	2.213147
**Age**				
1. 17 years or less (RC)				
2. 18 + years	1.22	0.114	0.9530528	1.9530528
**Religion**				
1. Christian (RC)				
2. Muslims	**1.35**	**0.031**	1.027105	2.027105
98. Others	1.43	0.224	0.8016927	1.8016927
**Migration Status**				
1. migrant (RC)				
2. Non migrant	0.93	0.54	0.7468912	1.7468912
**Level of education**				
1. None				
2. Primary	0.87	0.383	0.6464057	1.6464057
3. Secondary	**1.40**	**0.035**	1.024299	2.024299
**Type of employer**				
1. Self employed				
2. Employed	1.27	0.05	0.9999569	1.9999569
3. Parents and others	**1.65**	**0.024**	1.06847	2.06847
**Perceived fairness of payments**				
1. Yes				
0. No	**0.63**	**0.000**	0.5034684	1.5034684
**Has savings**				
0. No				
1. Yes	**1.45**	**0.001**	1.164592	2.164592
**Drinks alcohol**				
0. No				
1. Yes	**2.15**	**0.000**	1.571894	2.571894
**Physical violence during C-19**				
0 did not experience physical violence				
1. Experienced physical violence	0.92	0.654	0.647699	1.647699
**Sexual violence during C-19**				
0. did not experience				
1. Experienced violence	0.89	0.578	0.5827661	1.5827661
**Monthly earnings**				
Less than $70				
1. > = 70$	1.18	0.150	0.9416665	1.9416665
No earnings, not mentioned	**1.58**	**0.016**	1.091166	2.091166

^*^ Bold: P-Value less than 0.05.

The determinants of resilience are country, age, type of employer, perceived fairness of earnings, alcohol use, and whether a person has savings and monthly earnings. AGYW in Ghana had higher odds of resilience compared with Uganda (AOR 1.47; 95% CI 1.15–1.89); older AGYW compared with minors (AOR1.34; 95% CI 1.05–1.71), and employee or working with parents and others compared with those who are self-employed (AOR 1.35; 95% CI 1.07–1.72 for employee, and AOR 1.67; 95% CI 1.09–2.58 for parents and others). The odds were higher among those who had savings compared to those who had none (AOR 1.43, 95% CI 1.15–1.77), and those who were not earning had unspecified earnings compared to those who earned less than $70 USD. Those who perceived their payments to be unfair had reduced odds of resilience compared with those with fair payments (AOR 0.65; 95% CI 0.52–0.81). Surprisingly, AGYW who drank alcohol also had higher odds of resilience.

## Discussion

Results show that higher resilience was generally correlated with fair earnings, savings, secondary education, employment under parents or other relatives, and amongst AGYW in Ghana. First, supporting savings groups and financial literacy was identified as a key contributing factor to the resilience of AGYW in ASM communities. Results showed that although many participants indicated that they save money, many were not part of savings groups. However, having financial savings was one of the factors that correlated with higher resilience. As such, initiatives to support savings and savings groups should be encouraged. For example, in many sub-Saharan African countries, village savings and loan associations (VSLAs) are common to promote financial inclusion and women's empowerment (Karlan et al., 2013). Karlan et al. (2013) outline evidence that VSLAs improved resilience in Ghana, Uganda, and Malawi through food security and income generation. Adolescents in another part of this study also indicated that support for VSLAs and financial literacy was a priority for resilience. As such, continued support for VSLAs and financial literacy to encourage and empower adolescents to save can contribute to growing resilience amongst this vulnerable population.

Related to financial savings, fair and decent earnings were also indicated as a key aspect of normal to high resilience which aligns with the push for formalisation, integrating ASM into a formal regulation and oversight structure, in the scholarly literature and amongst NGOs. Formalisation has been a significant discussion in the ASM literature, pushing to create structures of accountability for governments and companies while also enforcing labour regulations and ensuring adolescents are compensated fairly (Fritz et al., 2017; Hilson & Osei, 2014; Maconachie, 2016). Formalising ASM would encourage improvement to working conditions and environmental regulations (Hilson et al., 2018).

Ghana's problematic ‘one-size-fits-all “small-scale” mining licence’ may serve as a case study for other countries in sub-Saharan African countries seeking to bring ASM operations under a legal and regulatory framework (Arthur-Holmes & Ofosu, 2024, p.6). Empowerment of women requires efforts on multidimensional fronts, including institutional support for women (e.g. access to mineral resources and financial assistance) and provision of different categories of mining licences that align with the financial capabilities and activities undertaken by women (Arthur-Holmes & Ofosu, 2024). Improving women's access to resources can also challenge gender norms by elevating women to prominent positions (e.g. cooperative leaders) within the ASM industry (Danielsen & Hinton, 2020). Boosting women's participation within the formal ASM space would increase the possibility for women in ASM to ‘have “female mining bosses”, who would be likely to provide better working conditions than “male mining bosses”’ (Arthur-Holmes & Ofosu, 2024, p.12). Evidently, by formalising the sector and increasing the opportunity for inclusion of women, greater regulation of wages and working conditions (Ofosu et al., 2022), higher resilience amongst AGYW would be supported through the opportunity to obtain fair, decent earnings. Insofar as formalisation and women's empowerment are concerned, there is scope for improved understanding of the thriving informal ASM economy to devise policies and tailor interventions that meet the diverse needs of women in the ASM sector who largely operate within the informal segment (Arthur-Holmes & Ofosu, 2024; Hilson et al., 2018). Designing specific policies and interventions for female ASM workers remains an ‘enormous’ challenge, one that requires ‘a more nuanced understanding of the region's informal economy itself, and the roles and trajectories of the women working here’ (Hilson et al., 2018, p. 311).

Employment under parents or other relatives was found to contribute to resilience. It is likely that employment under parents or relatives enabled AGYW to capitalise on existing social ties to navigate different types of challenges. This finding aligns with empirical evidence on women's successful negotiation for better remuneration with their male relatives who are ASM owners—highlighting how female ASM workers can use personal ties to their advantage (Arthur-Holmes et al., 2023a). Expectedly, results show that limited experience with sexual and physical violence correlated with normal to higher resilience which aligns with existing literature. Scholars note key factors that negatively impact resilience include environmental factors such as poverty, abuse, a lack of nurturing adults while growing, and experiencing physical and sexual violence (Asante, 2019; Levine, 2003; Namy et al., 2017). On the other hand, factors that contribute to higher resilience include healthy relationships, safety, opportunities for learning, and stability (Levine, 2003; Namy et al., 2017). As such, continuous engagement with diverse community stakeholders including parents, mentors, employers, community leaders, etc., through advocacy and raising awareness on the harms of physical and sexual violence can enhance the potential for healthy relationships, safety, and stability amongst adolescents. Scholars also encourage the need for policies and programmes to ensure that young people have access to education, services, and support through a whole-community approach to build resilience (Kabiru et al., 2012; Khanlou & Wray, 2014).

Finally, in Ghana, adolescents were identified to have higher resilience than in Uganda. This is likely due to the long-term work that has been completed in the Ghanaian ASM communities by NGOs such as the Northern Empowerment Agency (NEA). This project partnered with NEA to carry out the research data collection, analysis, and implementation of prioritised interventions within the communities. The Northern empowerment agency has worked in the Ghanaian ASM communities for over 40 years, building both health and social welfare programmes (Organisation, 2024). As such, although policies that support the safety and well-being of adolescents in mining exist both in Ghana and Uganda (Ghana Health Service, 2016; Government of Ghana, 2014; Ministry of Energy & Mineral Development, 2018; Ministry of Gender Labour Social & Development Uganda, 2012; Republic of Ghana, 2023), key to the resilience of this hard-to-reach population is long-term engagement and collaboration with the community to build context-specific, multi-stakeholder interventions (Chidwick et al., 2023). Multi-stakeholder collaboration is especially important due to the multiple vulnerabilities that ASM adolescents experience, such as limited earnings and lack of access to healthcare, which can lead to reduced resilience. Hence, holistic resilience building necessitates a multi-stakeholder approach (Chidwick et al., 2023).

Overall, most of these findings aligned with the literature; however, the finding that alcohol use is associated with normal to high resilience was contrary to the literature which links alcohol use to reduced resilience (Considine et al., 2017; Himmelsbach et al., 2023; Namy et al., 2017). While not disputing this claim, we asked participants about alcohol consumption rather than alcoholism or overuse. Participants who indicated they did consume alcohol could correspond with higher economic stability, as they were able to afford casual alcohol consumption. As such, these findings correlate with the related findings about savings and earnings, resulting in normal to higher resilience. However, further study is needed to further understand the connections between alcohol and adolescent girls' resilience in ASM.

## Limitations

We acknowledge two key limitations of this study. First, the difference in the language of resilience across countries and contexts may have introduced inconsistencies in interpretation. To mitigate this, we translated all the study tools into local languages and pre-tested tools to ensure that there was a common understanding of resilience for the study. Second, since the resilience theme was extracted from a larger survey study, there was no opportunity for the respondents to explain their responses, such as the meanings participants attached to their ‘bouncing back and or coping mechanisms’. However, a limited explanation is the nature of quantitative surveys. Since this is among the first studies to explore resilience in these contexts, the findings are relevant, informative, and could also inform qualitative discussions to further explore these meanings.

## Conclusion

Consistent with existing literature, our results show that higher resilience was generally correlated with fair earnings, savings, limited physical and sexual violence, secondary education, and, amongst adolescent girls in Ghana. These findings highlight the fact that although marginalised and last mile, the resilience scores and patterns, based on the widespread scale of participants involved, are similar to those of the general population. Findings also imply that strategies which have been used to build resilience amongst other populations including out-of-school adolescents and those living in informal settlements, may be relevant to the ASM population, although adaptation to context would be important. Targeted resilience interventions with the AGYW in ASM have the potential to break cycles of vulnerability from a life course perspective, whereby children are more resilient. Programmes should focus on savings and financial literacy, along with advocacy for mitigating sexual and gender-based violence and ensuring better access to education and services. Long-term multi-stakeholder collaborations are key to building resilience within ASM communities. Future research is encouraged to focus on qualitatively exploring what resilience, coping, and bouncing back mean to participants within their contexts, and the alcohol and resilience anomaly. There is scope for improved understanding of the specific needs of AGYW in the ASM sector in Ghana and Uganda. Understanding the context-specific needs of AGYW is critical for appropriate policy and programme development.

## Data Availability

Relevant data are included in the files of this manuscript.
